# Supplementary material for: MAPK Signaling Is Required for Generation of Tunneling Nanotube-Like Structures in Ovarian Cancer Cells
Source: Cancers (Basel). 2021 Jan 13;13(2):274. doi: 10.3390/cancers13020274 (PMC7828401; doi:10.3390/cancers13020274)
Supplement: Supplementary file 1 [file cancers-13-00274-s001.pdf]

Supplemental Materials: MAPK Signaling Is Required for Generation of Tunneling Nanotube-Like Structures in Ovarian Cancer Cells

Jennifer M. Cole, Richard Dahl and Karen D. Cowden Dahl

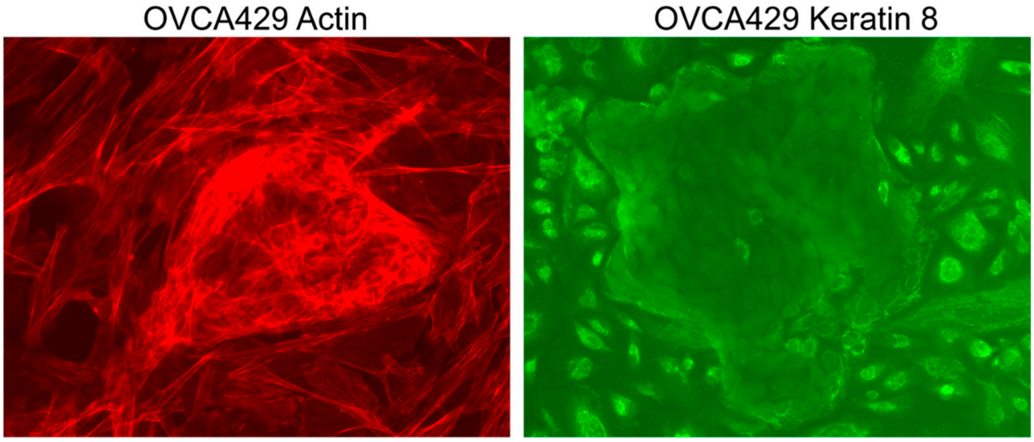

Figure S1. Ovarian cancer cells retain epithelial morphology when co-cultured with mesothelial. cells. OVCA429 cells were cultured on top of a confluent layer of primary human mesothelial cells and stained with Texas Red phalloidin after 24hours to denote F-actin based structures. OVCA429 maintain actin organization (Texas Red Phalloidin, left panel) and are surrounded by keratin 8 expressing mesothelial cells (immunofluorescence for Keratin 8, right panel). 20× Original magnification.

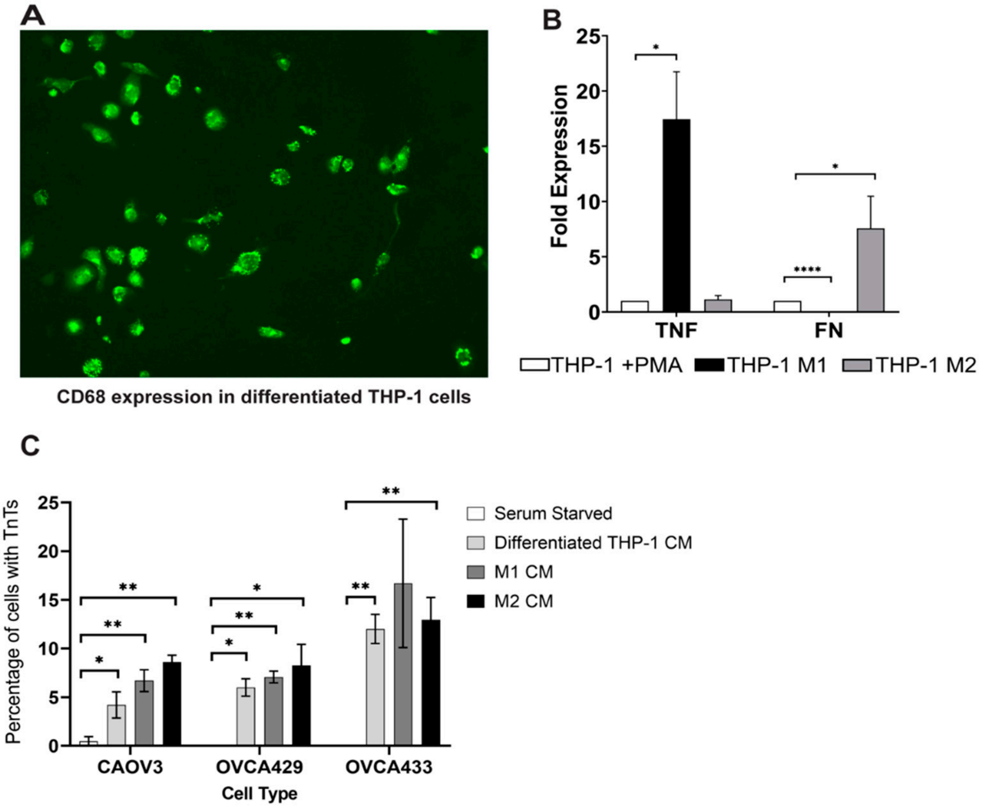

Figure 2. THP-1 macrophages are differentiated, polarized and induce TnTs in ovarian cancer cells. (**A**) THP-1 cells were differentiated using PMA. Immunofluorescence for CD68 on adherent cells

demonstrates successful macrophage differentiation of the THP-1 cells. (**B**) Differentiated THP-1 macrophages were differentiated with PMA and then left untreated or polarized to M1 or M2 macrophages. RNA was isolated and RT-qPCR was conducted for the M1 marker TNF and the M2 marker FN1. (**C**) Macrophage conditioned media-induced TnTs (as depicted in Figure 3) were quantitated based on outlined criteria. Total cell numbers and number of TnTs were counted for each field. Counts were averaged and error bars denote SEM between biological replicates. For statistics, comparisons are between the serum starved cells and CM treated. $N = 3$ $*p < 0.05$ $**p < 0.01$ $****p < 0.0001$.

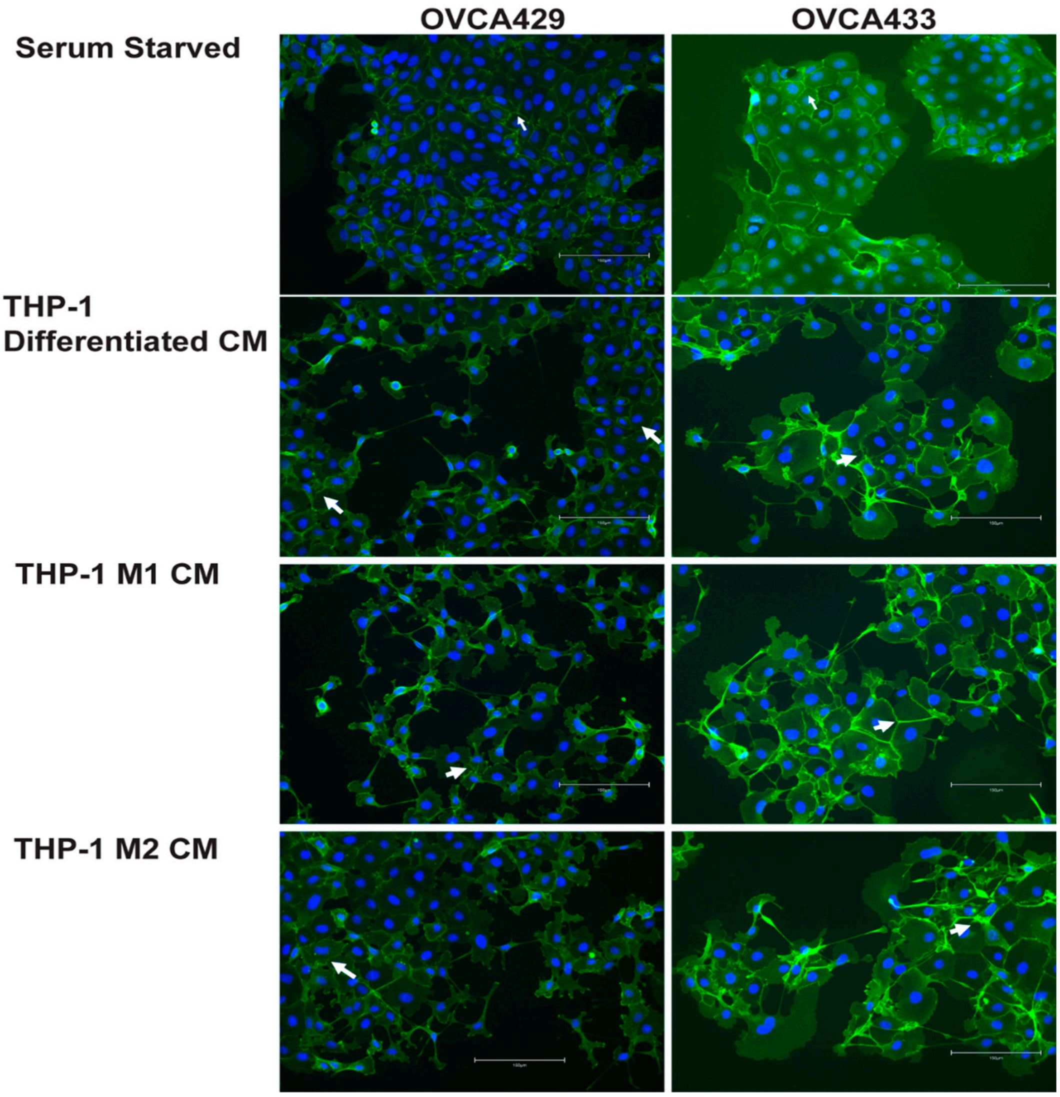

Figure S3. Expression of E-cadherin in ovarian cancer cells following exposure to macrophage. conditioned media. To determine if macrophage conditioned media promotes loss of E-cadherin or and EMT-like response cells were treated with macrophage conditioned media and immunofluorescence was conducted for E-cadherin. OVCA429 and OVCA433 cells were serum starved for 24 h and left untreated or treated with conditioned media from differentiated THP-1, M1, and M2 macrophages for an additional 24 h. Cultures were stained for E-cadherin (green) and DAPI (blue). 20× Original magnification. Scale bar = 150 µM. Arrow = E-cadherin junctions.

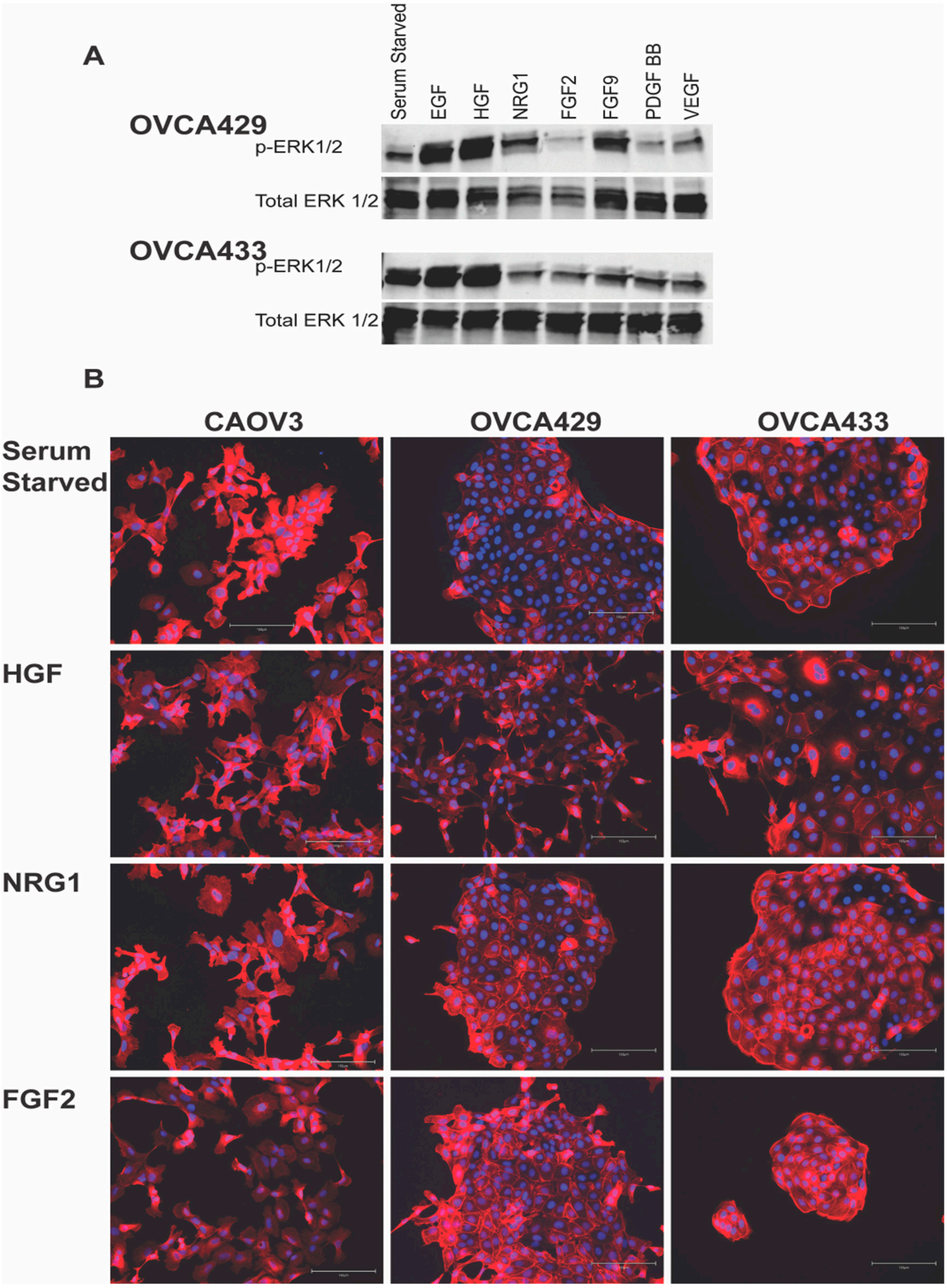

Figure S4. Treatment of ovarian cancer cells with growth factors. (**A**) OVCA429 and OVCA433. cells were serum starved for 24h and then treated with growth factors for 30min. Western blot was conducted for phosphorylated-p44/42 and total p44/42 to demonstrate that the MAPK signaling pathway was induced. (**B**) CAOV3, OVCA429, and OVCA433 cells were serum starved for 24h and then treated with HGF, NRG1 and FGF2 for an additional 24h. Cells were stained with Texas Red Phalloidin to visualize F-actin based structures and DAPI (blue). 20× Original magnification. Scale bar = 150 μM.

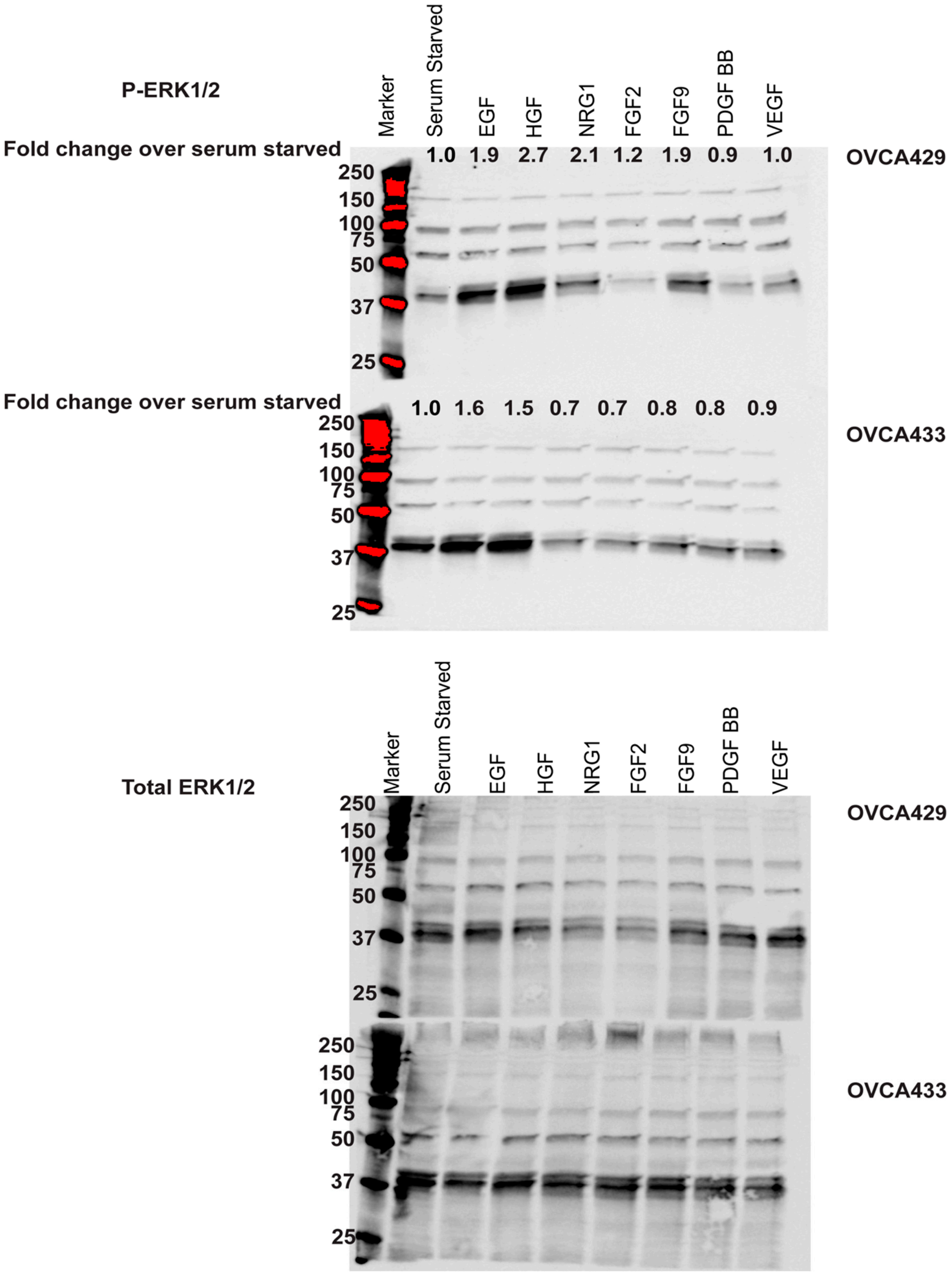

Figure S5. The western blots for phospho-p44/42 in Figure S4 were quantitated. Shown are the. uncropped western blots form Figure S4. The position of the molecular weight markers are shown. Additionally densitometry is shown for phospho-p44/42 and normalized to total p44/42. The densitometry is shown as a fold change over the serum starved condition.

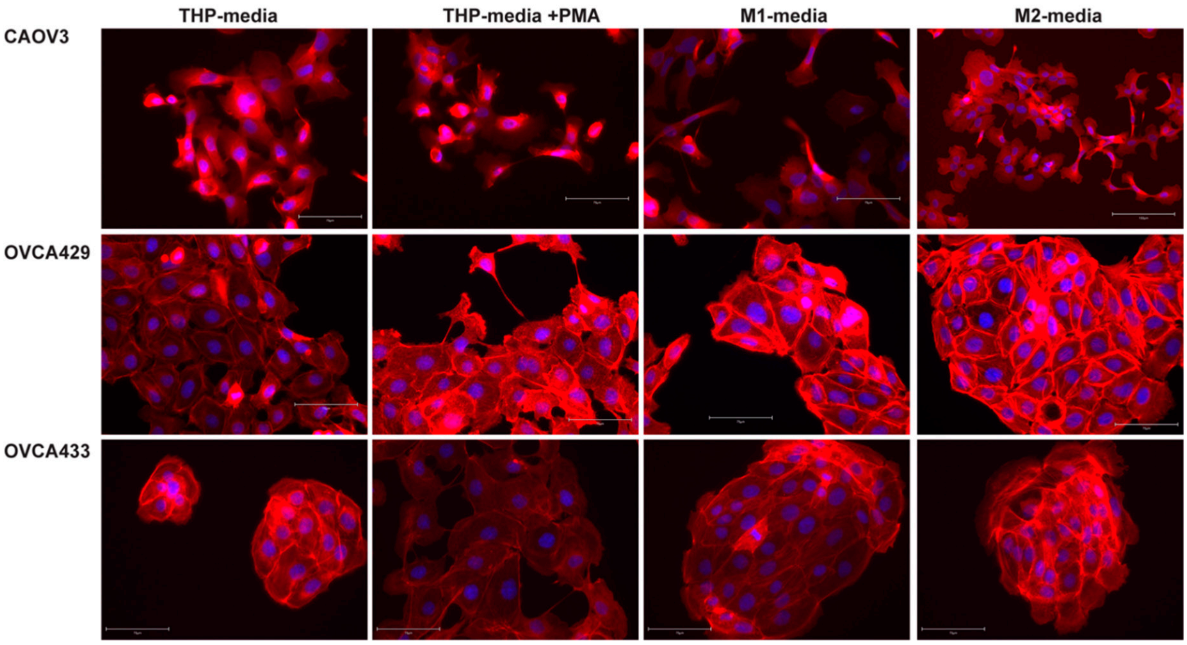

Figure S6. Impact of conditioned media controls on ovarian cancer cell morphology. CAOV3, OVCA429, and OVCA433 cells were serum starved for 24 h then treated with THP-1 media, THP-1 differentiation media (THP + PMA), M1 polarization (LPS and IFN) media or M2 polarization media (IL13 and IL4) for an additional 24h. Cells were stained with Texas Red Phalloidin to visualize F-actin based structures and DAPI (blue). 20× Original magnification. Scale bar = 150 μM.

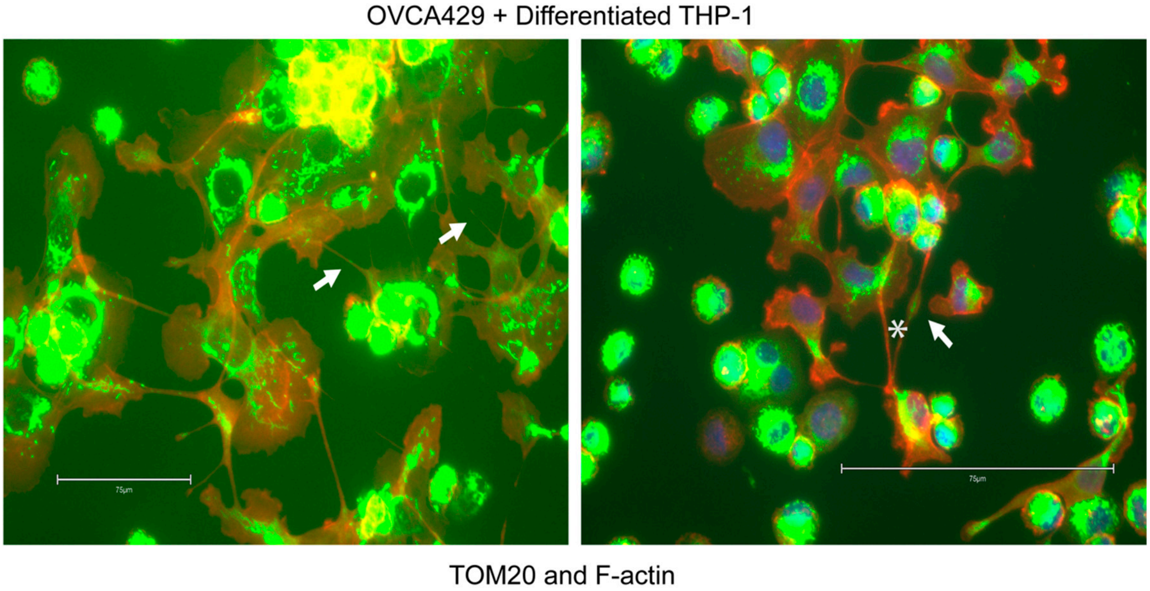

Figure S7. Mitochondria are present in OVCA429 membrane projections initiated by macrophages. To determine if mitochondria are transported via TnT and TnT-like projections in response to macrophages, we co-cultured differentiated THP-1 cells with OVCA429 cells. The cells were fixed and immunofluorescence was conducted for the mitochondira protein Tom20 and Texas Red Phalloidin was used to visualize F-actin based structures. 40× Original magnification. Scale bar = 75uM. The right panel is a zoomed in image. Arrow = TnT/membrane extension. * = thick membrane projection.

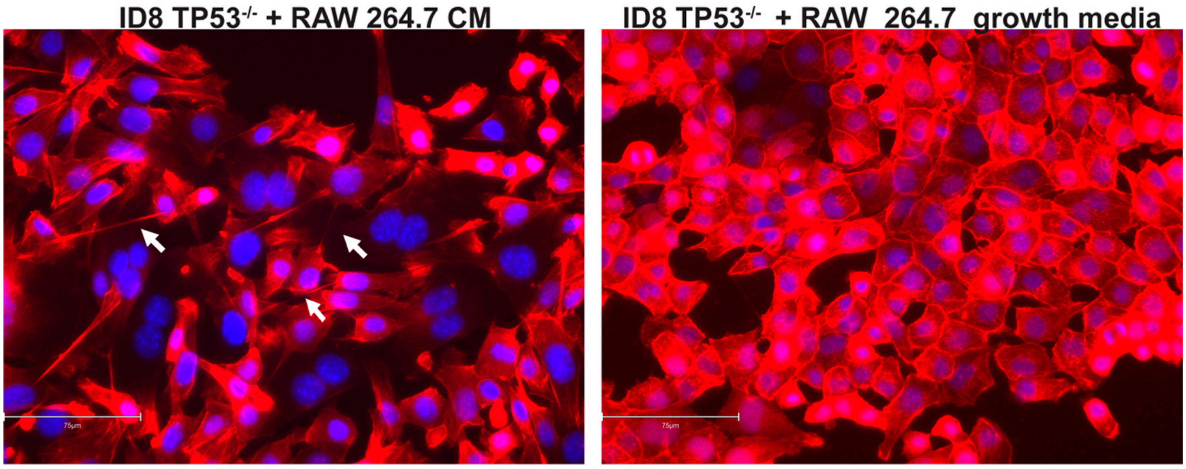

Figure S8. RAW 264.7 (mouse) macrophages induce TnTs in mouse ovarian cancer cells. To determine if a different source of macrophage conditioned media also induces TnTs in OCCs, we collected conditioned media from a mouse macrophage cell line and added it to a mouse ovarian cancer cell line. ID8 TP53-/-cells were cultured in the presence of RAW 264.7 conditioned media (CM) for 24 h or RAW 264.7 growth media (untreated control) for 24 hours. Cells were stained with Texas Red Phalloidin to visualize F-actin based structures and DAPI (blue). 40× Original magnification. Scale bar = 75 µM. Arrow = TnT/membrane extension.
